# Cataract as Early Ocular Complication in Children and Adolescents with Type 1 Diabetes Mellitus

**DOI:** 10.1155/2018/6763586

**Published:** 2018-03-20

**Authors:** Marko Šimunović, Martina Paradžik, Roko Škrabić, Ivana Unić, Kajo Bućan, Veselin Škrabić

**Affiliations:** ^1^Department of Pediatrics, University Hospital Centre Split, Spinčićeva 1, 21000 Split, Croatia; ^2^Department of Ophthalmology, University Hospital Centre Split, Spinčićeva 1, 21000 Split, Croatia; ^3^School of Medicine, University of Split, Šoltanska 2, Split, Croatia

## Abstract

Cataract is a rare manifestation of ocular complication at an early phase of T1DM in the pediatric population. The pathophysiological mechanism of early diabetic cataract has not been fully understood; however, there are many theories about the possible etiology including osmotic damage, polyol pathway, and oxidative stress. The prevalence of early diabetic cataract in the population varies between 0.7 and 3.4% of children and adolescents with T1DM. The occurrence of diabetic cataract in most pediatric patients is the first sign of T1DM or occurs within 6 months of diagnosis of T1DM. Today, there are many experimental therapies for the treatment of diabetic cataract, but cataract surgery continues to be a gold standard in the treatment of diabetic cataract. Since the cataract is the leading cause of visual impairment in patients with T1DM, diabetic cataract requires an initial screening as well as continuous surveillance as a measure of prevention and this should be included in the guidelines of pediatric diabetes societies.

## 1. Introduction

Approximately half a million children in the world today have type 1 diabetes mellitus (T1DM) with an estimation of 80,000 new cases every year [1]. T1DM and its complications are one of the biggest public health issues today and a leading cause of morbidity and mortality later in life [2]. Occurrence and cause of ocular complications comprising retinopathy, macular edema, papillopathy, cataract, glaucoma, strabismus, and refractive changes are well established in adults with T1DM [2, 3]. Unfortunately, there is only limited information about prevalence and pathophysiology of T1DM ocular complications in population of children and adolescents [3].

Even though cataract is one of the principal causes of visual deficiency in adult population with T1DM, it is a rare ocular complication at an early phase of T1DM in the pediatric population [4, 5]. In addition to a series of short-case reports with interesting clinical observations, to date, there are only several clinical studies on the characteristics and prevalence of early diabetic cataract in the pediatric population [3, 5–19]. The surgical solution continues to be a gold standard in the treatment of diabetic cataract, but the real challenge remains elucidating new therapeutic principles that would influence the pathophysiological mechanism of early diabetic cataract in the pediatric population [4, 20].

Our group was interested in finding clear recommendations for screening for early diabetic cataract in the pediatric population but was not able to find guidelines. This review article aims at summarizing all published findings about diabetic cataract as one of the first ocular complications of newly diagnosed T1DM in childhood and at emphasizing the importance of early screening. Our secondary objective is to clarify possible etiology and to highlight other conservative experimental therapeutic options for early cataract prevention and treatment in population of children and adolescents with T1DM.

## 2. Pathophysiology of Early Diabetic Cataract in Population of Children and Adolescents

The pathophysiological mechanism of early diabetic cataract has not been fully understood; however, there are many theories about the possible etiology of diabetic cataract early in childhood [14, 16]. Long-lasting hyperglycemia with consequential ketoacidosis and dehydration certainly plays an important role in the development of early diabetic cataract [5, 18]. Even though the majority of newly diagnosed pediatric patients with T1DM have aforementioned symptoms, only a small number of patients develop early diabetic cataract [16]. This observation certainly underlines the importance of other factors that influence the occurrence of early diabetic cataract including genetics, local factors, nutritional habits, and gender as well as growth and developmental changes in childhood [5, 15].

The activation of the polyol pathway under the influence of hyperglycemia and other cofactors is the most widely accepted hypothesis relating to the development of early diabetic cataract [4, 21]. A crucial enzyme in the cascade of polyol pathway is aldose reductase, which catalyzes reduction of glucose into sorbitol using NADPH prior to sorbitol reduction to fructose by sorbitol dehydrogenase with NAD^+^ as a cofactor [21, 22]. In experimental conditions, using various animal models, it has been demonstrated that increased sorbitol levels cause hyperosmolar conditions resulting in fluid retention due to an osmotic gradient disorder [4, 21, 22]. Osmotic damage is considered to be a very important factor in the development of early cataracts in childhood, leading to a change in the lens structure, gradual fibrosis, and, eventually, the formation of cataract [4, 5, 23]. However, it is important to note that earlier studies demonstrate that levels of sorbitol in human diabetic lens are not as high as in the animal models, so the exact role of osmotic damage during the formation of cataract in patients with T1DM still needs to be clarified [15, 24, 25].

Furthermore, several other metabolic pathways such as oxidative stress, activation of mitogen-activated protein kinase and cyclooxygenase-2, accumulation of cytosolic calcium, activation of NF-*κ*B, and activation of protein-1, which all relate to polyol pathway but signal through distinct mechanisms, were found important for the development of diabetic cataract [26–28]. The occurrence of oxidative stress plays a major role in gradual diabetic cataract development [26]. Reactive oxygen species (ROS) in diabetic patients are generated during oxidative stress in the process of advanced glycation end-product (AGEs) formation, but also as a byproduct of the polyol pathway due to accumulation of NADH and consequent NADH oxidase activity [21, 29]. The imbalance in antioxidant capacity results in increased availability of free radicals, which can also be associated with the possible formation of diabetic cataract [30].

In addition to this, a recent case report describes a patient with early diabetic cataract and an onset of monogenic-type diabetes caused by a mutation within the insulin gene (INS), which until now, has not been associated with diabetic cataract [31]. It is known that INS mutations are more frequent in patients with neonatal diabetes even though it is not entirely clear how this affects the formation of early diabetic cataract [31]. Altogether, these mechanisms have a certain impact on the occurrence of early diabetic cataract, but additional research is needed to unravel a clear pathophysiological pathway of early diabetic cataract in the pediatric population.

## 3. Prevalence and Other Clinical Study Features of Early Diabetic Cataract in Population of Children and Adolescents

The prevalence of early diabetic cataract in the population of children and adolescents depending on the authors varies between 0.7 and 3.4% [3, 5, 9, 10, 15, 32]. The highest prevalence has been described in the study from 1985, but these results might be explained by the substandard regulation of T1DM at the time and fewer therapeutic approaches [32]. Majority of the new studies reported the prevalence of approximately 1%, but a recent study in USA by Geloneck et al. reported a significantly higher prevalence of 3.3% [3, 9, 15]. This may be due to a slightly longer duration of T1DM before initial cataract diagnosis, but future studies including larger patient cohorts and multinational cooperation will fully clarify the precise prevalence in population [3]. However, the occurrence of cataract in most patients is the first sign of T1DM or it occurs within 6 months of diagnosis of T1DM, which indicates the importance of early screening. Wilson et al. note that T1DM should be considered in cases with acquired cataract of unknown etiology following cataract extraction, particularly in the younger patients [5].

In addition to this, majority of studies assessing prevalence of cataract in T1DM were performed in developed countries, so it is not entirely possible to exclude the influence of various environmental factors and socioeconomic status of the patients on the prevalence of early ocular T1DM complications including cataract. Summary of clinical and individual characteristics of patients that have been published in articles and case reports is shown in Table 1. The youngest patients described in the literature with early diabetic cataracts were 5 years old, but most of the patients were adolescents [5, 12, 15]. Iafusco et al. reported equal gender distribution in T1DM pediatric population with early diabetic cataract, while most of other authors had significantly more female patients [5, 9, 15]. The same group of authors describes the appearance of ketoacidosis as a sign of decompensated T1DM in all patients, which is in accordance with majority of older publications [15]. Interestingly, more recent findings suggest a smaller occurrence of ketoacidosis [17, 18]. Furthermore, there is a significant variability regarding the level of hemoglobin A1c (HbA_1c_) at the diagnosis of diabetes itself, but also at the onset of early diabetic cataracts [3, 8, 9]. Iafusco et al. pointed out in their study that for each percentage point from 12.8 to 14.1% of HbA_1c_ level, appearance of early diabetic cataract increases 3.6 times [15]. Altogether, these findings highlight the importance of good control of glycemia and HbA_1c_ levels as one of the risk factors for development of early diabetic cataract.

Regarding morphology of early diabetic cataract, Wilson et al. reported multiple morphologies including posterior subcapsular, lamellar, cortical, snowflake, and milky white type of early diabetic cataract, while most of other authors discriminate fewer types with posterior subcapsular cataract described as the most common type of diabetic cataract in childhood [5, 9, 14, 17, 18].

## 4. Prevention and Treatment of Early Diabetic Cataract in Population of Children and Adolescents

Typical early symptoms of T1DM such as polyuria, polydipsia, polyphagia, and weight loss need to be recognized as soon as possible thus reducing the exposure of the lens to hyperglycemia and other consequences of severe metabolic conditions, which altogether can have a positive impact on the formation of early diabetic cataracts in the pediatric population [5, 33]. American Diabetes Association (ADA) and the International Society for Pediatric and Adolescent Diabetes (ISPAD) as two major associations of pediatric diabetologists provide comprehensive guidelines for the prevention, diagnosis, and treatment of T1DM [34, 35]. Interestingly, ADA did not include any recommendation about screening for early diabetic cataract, although it is endorsed that the first ophthalmological examination for assessment of retinopathy should be done when the patient reaches the age of 10 years, after puberty occurs, or when the duration of T1DM is longer than 3 to 5 years [35]. In addition to this, ISPAD guidelines recommend that an initial eye examination should be considered in order to detect early diabetic cataract or major refractive errors, but there are no clear further instructions about extension of screening for diabetic cataract in population of children and adolescents [34].

In the past two decades, phacoemulsification is the most common technique of cataract extraction in the developed world [36]. Types of surgery differentiate between younger and older children. Attributable to soft cataract in younger children, use of phacoemulsification is not mandatory, whereas older children and adolescents should proceed to phacoemulsification [20, 37]. Most of the patients operated from 1982 underwent either intracapsular cataract extraction (ICCE), extracapsular cataract extraction (ECCE), or phacoemulsification surgery. Geloneck et al. reported that only 5 out of 12 of their patients had visually significant cataract and underwent cataract surgery [3]. However, cataract surgery is not without complications, and it is especially necessary to take into account the risks of long-term T1DM and the effects on growth and development of anterior eye segment [38]. The most common complications after cataract surgery are posterior capsular opacification (PCO), secondary glaucoma, retinal detachment, amblyopia, and acute complications (incision leakage, increased intraocular pressure, edema, and uveitis) [38, 39]. There are differences in management of PCO between younger and older children, as PCO occurs more often in younger children. It is generally advised that primary posterior capsulorhexis (PPC) should be performed in children younger than 4 years of life, since the risk of developing PCO even if posterior capsule remains intact is 100%, due to more reactive inflammatory response in younger age [20, 39, 40]. Even after PPC is performed, there is a substantial risk of secondary visual axis opacification (VAO) due to migration of lens epithelial cells from anterior vitreous; thus, it is recommended to perform anterior vitrectomy (AV) together with PPC in infants and young children [20, 41]. There are no clear guidelines whether PPC should be combined with AV in older children, which could be of great importance in children with diabetic cataract. Whitman and Vanderveen suggest that older children with simple PCO can undertake laser capsulotomy and those with both PCO and VAO can proceed to surgery [42]. Randomized controlled study in 27 children aged between 4 and 14 years who underwent the intervention of cataract surgery with or without PPC and AV demonstrated better visual acuity and significantly less PCO in the group that undergone cataract surgery with PPC and AV [39, 43]. Elkin et al. revised the incidence of PCO in all age groups of pediatric cataract patients who underwent cataract extraction followed by IOL implantation without PPC and AV and found occurrence of PCO up to 90% [44]. Khaja et al. shortly reported that 233 eyes of children younger than 18 with cataract that underwent cataract extraction followed by implantation of either *AcrySof* 1-piece lens (SN60AT) or 3-piece lens (MA60AC) without PPC and AV and had statistically higher incidence of VAO compared to groups with the same lens implantation that received PPC and AV, implicating that prospective study with longer follow-up should be performed in order to illuminate impact of VAO [45]. Additionally, few authors reported that cataract surgery had influenced the onset and progression of retinopathy [4]. Falck and Laatikainen demonstrated that out of 11 eyes that were surgically treated in pediatric patients with early diabetic cataract, only 3 eyes did not develop retinopathy after 8 years of follow-up [10]. Another important problem is the choice of an appropriate intraocular lens, which would provide adequate control of the posterior segment of the eye and possible need for laser treatment or vitrectomy in patients with progression of retinopathy [2, 5]. Despite the careful preoperative measurement of the eye, calculation of power of the IOL, and prediction of refractive outcome, refractive error can occur in adult age due to emmetropization of the eye [46]. The majority of published articles about calculation of IOL power refer to surgical approach to congenital cataracts in infants [47]. According to the case report describing the youngest T1DM patient with early diabetic cataract at the age of 5, even though risk for development of amblyopia is reduced, eye growth is still not finished and probability of refractive error persists. Eye growth after 18 months of life, in juvenile age, enters a slow phase of 0.01 mm in diameter per month and myopic shift occurs; therefore, targeted IOL power should be undercorrecting (hyperopic) in order to ensure emmetropia or low myopia in adult age [41, 48–50]. Consequently, long-term follow-up of patients with diabetic cataract is needed to understand possible influence of surgical treatment on the development of ocular complications.

A small number of studies have shown gradual regression and resolution of diabetic cataracts in the pediatric population. Jin et al. reported two cases of reversible cataract that gradually disappeared over several months with good glycemic control [17]. Phillip et al. suggested that duration of T1DM symptoms prior to therapy has a key role in the reversibility of diabetic cataract [6].

Today, there are many experimental therapies for the treatment of diabetic cataracts, but most are at the stage of laboratory-level studies; however, only several have been tested in clinical trials, but there is no information specific for the pediatric population. Previously, studies have been published describing various aldose reductase inhibitors that clearly prevent the onset of cataract on induced diabetic mouse model, but unfortunately most of them have numerous side effects [22]. Recently, research focus has been shifted to unrefined nutrients extracted from plants, teas, and fruits, which inhibits aldose reductase [22, 51]. Other possible preventive supplements are also described in the literature including nutritional antioxidants such as pyruvates and vitamins C and E, but further studies are needed to fully clarify their role [52]. A positive effect of hyperbaric conditions on lowering the glucose level and delay of cataract onset in diabetic mouse model has also been described and it is assumed that it is related to inhibition of aldose reductase and other mechanisms of oxidative stress [30].

## 5. Conclusion

Early diabetic cataract, although a rare complication of T1DM in the pediatric population, requires an initial screening as well as continuous surveillance as a measure of prevention since it is the leading causes of visual impairment in pediatric T1DM patients, and this should be included in the guidelines of major pediatric diabetes societies. The prevention of long-term hyperglycemia and rapid implementation of intensive insulin therapy certainly reduce the prevalence of early diabetic cataract in children and adolescents. Furthermore, additional studies are needed to thoroughly explain the etiological cause and therefore improve the prevention and treatment of diabetic cataract in population of children and adolescents.

## Figures and Tables

**Table 1:** Characteristics of studies and patients with early diabetic cataract in population of children and adolescents.

Authors	Country	Age at cataract diagnosis (yrs)	T1DM duration at diagnosis (yrs)	HbA_1c_ at T1DM diagnosis (%)	Mean HbA_1c_ (%)	Morphology of cataract	Gender female/male	Surgical treatment	Number of patients
Phillip et al. [6]	USA	14	0	17	/	PSC	1/0	0	1
Alouf and Pascual [7]	USA	9	0	22.2	/	S	1/0	0	1
Datta et al. [8]	UK	11 to 14	0 to 1	15.1 to 21.2	/	PSC, S, C, D	3/2	4	5
Montgomery and Batch [9]	Australia	9 to 16	0 to 13	7.2 to 15.2	6.5 to >14	PSC, C	8/1	8	9
Falck and Laatikainen [10]	Finland	9.1 to 17.5	0 to 3.9	/	/	PSC, S, DI	5/1	6	6
Awan et al. [11]	UK	18	0	10.5	/	C, D	0/1	1	1
Wilson et al. [5]	Multiple	5 to 16.5	/	/	/	Multiple	11/3	12	14
Costagliola et al. [12]	Italy	5.3 to 13.2	0 to 0.1	12.8 to 14.5	/	PSC, S, D, DI	0/3	3	3
Patel et al. [13]	USA	10	0	17.9	13.4	PSC, C, DI	0/1	1	1
Skrabic et al. [14]	Croatia	16.8	0.2	15.5	11.7	PSC	1/0	1	1
Iafusco et al. [15]	Italy	5.5 to 15	0 to 0.2	12.8 to >14	8 to 11.6	PSC, S, D, DI	3/3	5	6
Uspal and Schapiro [16]	USA	13	0	>14	/	D	1/0	1	1
Jin et al. [17]	China	9 to 11	0	30 to 31	14.7 to 20.4	PSC, S, C, D	2/0	0	2
Goturu et al. [18]	UK	13.3	0.3	16.6	11.6	PSC	1/0	1	1
Geloneck et al. [3]	USA	7.5 to 18	0 to 15	7.7 to >14	7.3 to >14	PSC, C, D	/	5	12
García García and García Robles [19]	Spain	12 to 13	0.5 to 10	14 to 14.5	8.7 to 10	S, C, DI	1/1	1	2

Yrs: years; T1DM: type 1 diabetes mellitus; HbA_1c_: hemoglobin A1c; PSC: posterior subcapsular; S: snowflake; C: cortical; D: dense; DI: diffuse.
